# Mental Health Challenges Faced by Young Medical Professionals in Iran: A Closer Look at the Rising Suicide Rates

**DOI:** 10.31661/gmj.v13i.3398

**Published:** 2024-06-22

**Authors:** Farahnaz Azizi, Fatemeh Kafami Ladani, Ehsan Jangholi, Mohammad Rahimi, Roya Dokoohaki, Kamkar Aeinfar

**Affiliations:** ^1^ Department of Midwifery, Astara Branch, Islamic Azad University, Astara, Iran; ^2^ Nursing Care Research Center, Iran University of Medical Sciences, Tehran, Iran; ^3^ Department of Neurosurgery, Shariati Hospital, Tehran University of Medical Sciences, Tehran, Iran; ^4^ Brain and Spinal Cord Injury Research Center, Neuroscience Institute, Tehran University of Medical Sciences, Tehran, Iran; ^5^ Student Research Committee, School of Medicine, Mazandaran University of Medical Sciences, Mazandaran, Iran; ^6^ Community-Based Psychiatric Care Research Center, Nursing and Midwifery School, Shiraz University of Medical Sciences, Shiraz, Iran; ^7^ Biomedical Innovation and Start-up Student Association (Biomino), Tehran University of Medical Sciences, Tehran, Iran

**Keywords:** Mental Health, Suicide, Medical Professionals

## Dear Editor,

The recent report of the tragic suicide of a young Iranian cardiologist and a genius rheumatologist during the last months has shocked the medical community and highlighted the urgent need to address the mental health crisis faced by young medical professionals in Iran.

According to the reports and statistics of the Islamic Republic of Iran Medical Council, the number of suicides in 2023 was 16 cases, which, compared to the general population, shows an increase of 40 and 130 percent among male and female physicians, respectively [1]. Another statistic shows that the suicide rate of physicians has increased to five-fold in the last few years. On the other hand, during the first two months of the Iranian new year (March 20 to May 20), 10 doctors have committed suicide, which is a very alarming statistic [1]. As we are faced with the consequences of these losses, there is a need to pay attention to the systemic issues that contribute to the rising suicide rate among young medical professionals in Iran.

In recent years, Iran has seen an alarming trend of increasing suicide rates among health workers, particularly young professionals [2]. The demanding nature of the medical profession, combined with social stigma, lack of mental health support, and burnout led to many mental health challenges for these individuals [3].

Indeed, young medical professionals in Iran are subjected to significant pressure to excel in a highly competitive field, often at the expense of their mental well-being [4]. Constant financial demands, long working hours, exposure to trauma, and fear of making mistakes create a stressful environment that can lead to anxiety, depression, and emotional exhaustion [5].

Furthermore, the stigma surrounding mental health in Iran, especially within the medical community, presents a major barrier to seeking help for psychological distress [6]. The fear of being judged or labeled as incompetent prevents many young professionals from accessing the support they need to cope with their mental health challenges [7]. Hence, breaking down these barriers and fostering a culture of openness and acceptance are essential steps for creating a safe environment in which individuals can address their emotional well-being.

Despite the growing awareness of mental health issues, there remains a critical need for mental health support and resources for young medical professionals in Iran. Limited access to counseling services, insufficient training in mental health literacy, and inadequate mental health professionals further exacerbate these challenges [8].

The demanding schedules and long hours of healthcare workers contribute to burnout, a state of emotional, physical, and mental exhaustion that can have serious consequences, including suicidal thoughts [9]. Consequently, recognizing the signs of burnout and implementing interventions to support the mental well-being of young professionals is crucial in preventing further tragedies such as the recent suicide of an Iranian cardiologist. This includes providing counseling services, access to mental health professionals, and implementing regular mental health check-ups [9]. Lastly, collaboration among medical associations, government agencies, and educational institutions is essential in addressing this crisis. Indeed, by working together, they can develop and implement policies that prioritize the mental well-being of young medical professionals and prevent future tragedies [10].

In conclusion, by recognizing the impact of work-related pressures, overcoming social stigma, improving access to mental health resources, identifying the signs of burnout, and promoting mental health awareness, we can create a more supportive and empathetic environment for these individuals. Actually, by prioritizing the mental well-being of medical staff and ensuring that they receive care and support, they can better navigate the challenges of their profession.

## Acknowledgment

We are grateful to Dr. Reza Azizian, CEO and co-founder of the Biomedical Innovation and Sturt-Up Student Association (Biomino), Tehran University of Medical Sciences for his cooperation and scientific assistance. Also, we thank to Dr. Milad Shafizadeh at Department of Neurosurgery, Shariati Hospital, Tehran University of Medical Sciences, Tehran, Iran for his supports.

## Conflict of Interest

The authors declare that there was no any conflict of interest. Also, one of the authors of the article (Ehsan Jangholi) is the deputy editor of the GMJ journal. Based on the journal policy, he was completely excluded from any review process of this article, as well as the final decision.
